# Associations between anthropometric indicators and refraction in school-age children during the post-COVID-19 era

**DOI:** 10.3389/fpubh.2022.1059465

**Published:** 2023-01-18

**Authors:** Wenzheng Du, Gang Ding, Xiying Guo, Kadiya Abudukeyimu, Yanzhu Wang, Lijun Wang, Xiaoli Qi, Yuxian Ning, Ning Hua, Linlin Song, Xue Li, Jing Li, Ying Zhang, Nan Wei, Xuehan Qian

**Affiliations:** ^1^Tianjin Key Laboratory of Retinal Functions and Diseases, Tianjin Branch of National Clinical Research Center for Ocular Disease, Eye Institute and School of Optometry, Tianjin Medical University Eye Hospital, Tianjin, China; ^2^Tianjin Beichen Traditional Chinese Medicine Hospital, Tianjin, China

**Keywords:** myopia, refraction, anthropometric indicators, associations, school-age children

## Abstract

**Purpose:**

To explore the associations between anthropometric indicators and refraction in school-aged children in the post-COVID-19 era.

**Methods:**

Data were collected from 25,644 children aged 7 to 12 years in 48 elementary schools in Tianjin. The comprehensive examination included height, weight, systolic blood pressure (SBP), diastolic blood pressure (DBP), refraction, and calculation of BMI, with a follow-up visit after 6 months. Myopia was defined as spherical equivalent refraction (SER) ≤-0.50 diopter (D). Bivariate correlation coefficients and multiple linear regression models were used to explore the cross-sectional and longitudinal associations between anthropometric indicators (height, weight, BMI, SBP, and DBP) and refraction.

**Results:**

The mean changes in height, weight, BMI, SBP, DBP, and SER of the participants were 4.03 ± 2.18 cm, 3.10 ± 2.39 kg, 0.45 ± 1.16 kg/m^2^, 2.26 ± 14.74 mmHg, 2.18 ± 11.79 mmHg and −0.17 ± 0.51 D, respectively. Overall, height, weight, BMI, SBP, and DBP were all correlated with SER (*r* = −0.324, *r* = −0.234, *r* = −0.121, *r* = −0.112, *r* = −0.066, both *p* < 0.001), and changes in height and weight were correlated with changes in SER (*r* = −0.034, −0.031, both *p* < 0.001). Furthermore, multiple linear regression analysis revealed that the association of BMI, SBP, and DBP with SER was significant in myopic children but not in non-myopic children. The association between changes in weight and changes in SER was only present in non-myopic children but not in myopic children.

**Conclusion:**

Height and weight were negatively correlated with SER in both cross-sectional analysis and longitudinal changes, indicating that children's height, weight and growth rate may be used as a reference indicator for myopia risk prediction and myopia progression monitoring.

## Introduction

In recent decades, the prevalence of myopia has rapidly increased (1). According to projections, nearly half of the world's population will suffer from myopia by 2050 (2). Among children and adolescents, myopia is also showing a high prevalence. Within East and Southeast Asia, including China, Korea, and Singapore, myopia rates among school-age children are significantly higher than in other parts of the world (3). Myopia's high prevalence severely impacts people's physical and mental health (4). The etiology of myopia has not yet been fully understood, so there is an urgent need to discover the key factors affecting the formation of early myopia through the study of refractive development patterns and then discover effective prevention methods and intervention measures.

As we considered the etiology of myopia, we noted that anthropometric indicators, such as height, were thought to be related to refraction. Previous studies consistently show that height positively correlates with eye axis length (AL) (5, 6). Nevertheless, there is no consensus on the relationship between height and refraction. In addition, some studies report that people with higher body mass index (BMI) are more likely to be myopic (7), but some studies did not find this association (8). In brief, previous studies on the correlation between anthropometric indicators and refraction have not reached consistent conclusions.

The outbreak of a new coronavirus disease (COVID-19) in December 2019 has affected many aspects of people's lives. The Chinese government started closing schools and providing distance education for children nationwide in late January 2020 as an emergency measure to prevent the spreading of the infection. As China entered the post-COVID-19 era, most schools were gradually reopened from August to September 2020. Despite the effectiveness of the overall epidemic prevention efforts, there are still sporadic and recurring outbreaks in some places, which has promoted appropriate adjustments to campus epidemic prevention measures. Usually, campuses act relatively loosely against the epidemic; conversely, campuses will immediately be on alert if the pandemic has signs of resurgence and take anti-epidemic actions such as closing schools.

The COVID-19 pandemic has profoundly affected children's daily life, including insufficient physical activity, excessive sedentary behavior, and unbalanced diets (9, 10). School closures associated with COVID-19 may affect children's physical growth and weight changes (11, 12), and may also accelerate the change of their refraction toward myopia (13, 14). Nearly all previous studies on the relationship between anthropometric indicators and refraction were conducted prior to the COVID-19 outbreak, and they were all cross-sectional in design. A longitudinal set of data is needed to understand the relationship between them during the post-COVID-19 era. Myopia commonly occurs in children during their early school years and increases in magnitude as they age (15). Therefore, it is most appropriate to study the effect of physical growth on the refractive development of growing students. In this study, we explored the associations between anthropometric indicators, including height, weight, BMI and blood pressure and refraction in children aged 7–12 years in the post-COVID-19 era in China.

## Methods

### Study design and population

This school-based study was approved by the Ethics Board of Tianjin Medical University Eye Hospital. Informed written consent was obtained prior to the start of the study from the parents of all participants according to the Declaration of Helsinki. Anthropometric indicators, including height, weight, BMI and blood pressure, and refraction screening were performed on two consecutive occasions from April to June 2021 and from October to December 2021 in 48 elementary schools in Beichen District, Tianjin, China. All students aged 7–12 were invited, but participation in this study was voluntary. Students without parental consent and those with amblyopia, heterotropia or any ocular or systemic pathologies were excluded. In total, 31,068 children were recruited, and 25,644 (82.5%) children successfully completed two examinations.

### Refraction screening

Non-cycloplegic refractive error was tested using the Spot™ vision screener (Welch Allyn, Skaneateles Falls, NY). Testing was conducted by trained staff who obtained results from each child in three trials. During the test, the examiner asks the subject to look at the device binocularly from a one-meter distance. Red reflex images are acquired from the subject, and non-cycloplegic refractive status, pupil size and gaze deviation are automatically recorded. The device will flag a referral for a complete eye examination if significant refractive error, anisometropia or strabismus are detected. All screened subjects from this study were successfully tested. The measurement range of the Spot screener was limited to ± 7.50 D. If the refraction was out of range, ± 8.00 D was recorded for further analysis. The child's spherical equivalent refraction (SER) is recorded automatically for both eyes. Myopia was defined as an SER of −0.50 D or less.

### Anthropometric measurements

Height and weight were measured by removing heavy clothing and standing barefoot on a calibrated electronic height and weight meter, with the medical staff holding the measuring scale firmly over the subject's head and recording the readings in centimeters (cm) and kilograms (kg), respectively after they had stabilized. BMI was calculated as weight/height and recorded in kilograms per square meter (kg/m^2^). The systolic blood pressure (SBP) and diastolic blood pressure (DBP) were using an automated device (OMRON HEM-7136). The measurement was taken in the seated position with the right arm supported at heart level after at least 2 min rest and recorded in millimeters of mercury (mmHg).

### Statistical analysis

All statistical analyses were performed with the SPSS (IBM Corp. Released 2012. IBM SPSS Statistics for Windows, Version 21.0. Armonk, NY: IBM Corp.) and *p* < 0.05 were considered statistically significant. As the biometric data for the right and left eye were highly correlated, analyses were performed using data for the right eye only. Participants were classified into myopic and non-myopic groups based on their refractive status at baseline examination. The difference between the second and baseline examinations is defined as changes. Descriptive statistics of changes in SER and anthropometric indicators were calculated. *T*-tests were performed for quantitative variables, and Chi-square tests were performed for categorical variables to analyse the differences in basic characteristics between the two examinations and between the two groups of participants.

Bivariate correlations between SER and anthropometric indicators were calculated. Linear regression models were constructed to assess the effects of anthropometric indicators (as independent variables) on refraction (as dependent variables). Tests for linear trends were performed by entering the median value of each category of the anthropometric indicator based on quartiles as a continuous variable into the models. Multiple linear regression models were fitted separately to participants with and without myopia to assess the effect of anthropometric indicators on refraction for different refractive states. The relationship between anthropometric indicators changes and refraction changes were then analyzed according to the method described above.

## Results

A total of 31,068 children were recruited for this study. Five hundred twenty-four children were not examined because they had ocular or systemic diseases or were not cooperative for personal reasons. Four thousand nine hundred children did not complete follow-up examinations due to graduation, school changes, or other reasons. The remaining 25,644 (82.5%) children aged 7–12 (mean = 9.35 ± 1.51) years completed two examinations, consisting of 13,308 (51.9%) males and 12,336 (48.1%) females. At the final examination, there were significant differences in the SER, height, weight, BMI, SBP, and DBP compared to the first examination (paired *t*-test, both *p* < 0.001). The overall prevalence of myopia increased from 35.17% (9,020 of 25,644) to 39.78% (10,200 of 25,644), with significant differences (Chi-square test, *p* < 0.001). Table 1 presents the demographic characteristics of the analysis cohort by myopic or non-myopic at baseline. Myopic participants were taller, heavier, had a larger BMI, and had higher SBP and DBP. These individuals grew faster in height, gained more weight, and had more remarkable changes in SBP. Moreover, myopic children had more negative refraction and had greater myopic shifts than non-myopic children (both *p* < 0.001).

**Table 1:** Summary of the characteristics of the participants.

	**Total**	**Myopic**	**Non-myopic**	P^*^value
Age (years)	9.35 ± 1.50	10.04 ± 1.36	8.97 ± 1.45	<0.001
SER (D)				
Baseline	−0.53 ± 1.44	−1.98 ± 1.44	0.26 ± 0.56	<0.001
After 6 months	−0.69 ± 1.57	−2.28 ± 1.54	0.17 ± 0.63	<0.001
Δ SER	−0.17 ± 0.51	−0.30 ± 0.65	−0.10 ± 0.40	<0.001
Height (cm)				
Baseline	139.16 ± 11.12	143.92 ± 10.57	136.58 ± 10.54	<0.001
After 6 months	143.19 ± 11.39	148.04 ± 10.80	140.56 ± 10.82	<0.001
Δ Height	4.03 ± 2.18	4.12 ± 2.22	3.98 ± 2.16	<0.001
Weight (kg)				
Baseline	36.87 ± 12.46	40.56 ± 12.97	34.87 ± 11.69	<0.001
After 6 months	39.97 ± 13.35	43.93 ± 13.79	37.81 ± 12.59	<0.001
Δ Weight	3.10 ± 2.39	3.37 ± 2.51	2.95 ± 2.31	<0.001
BMI (kg/m^2^)				
Baseline	18.60 ± 4.07	19.20 ± 4.23	18.27 ± 3.94	<0.001
After 6 months	19.05 ± 4.14	19.67 ± 4.27	18.71 ± 4.02	<0.001
Δ BMI	0.45 ± 1.16	0.47 ± 1.16	0.44 ± 1.16	0.057
SBP (mmHg)				
Baseline	105.52 ± 12.89	107.18 ± 12.86	104.62 ± 12.82	<0.001
After 6 months	107.78 ± 13.70	110.12 ± 13.82	106.51 ± 13.47	<0.001
Δ SBP	2.26 ± 14.74	2.94 ± 14.68	1.89 ± 14.76	<0.001
DBP (mmHg)				
Baseline	67.13 ± 9.41	67.91 ± 9.36	66.70 ± 9.41	<0.001
After 6 months	69.31 ± 9.99	70.17 ± 9.95	68.85 ± 9.99	<0.001
Δ DBP	2.18 ± 11.79	2.25 ± 11.70	2.14 ± 11.84	0.484

Data are presented as the means with SD. SER, spherical equivalent refraction; BMI, body mass index; SBP, systolic blood pressure; DBP, diastolic blood pressure.

* Determined using independent-samples t-test.

**Figure 1:**
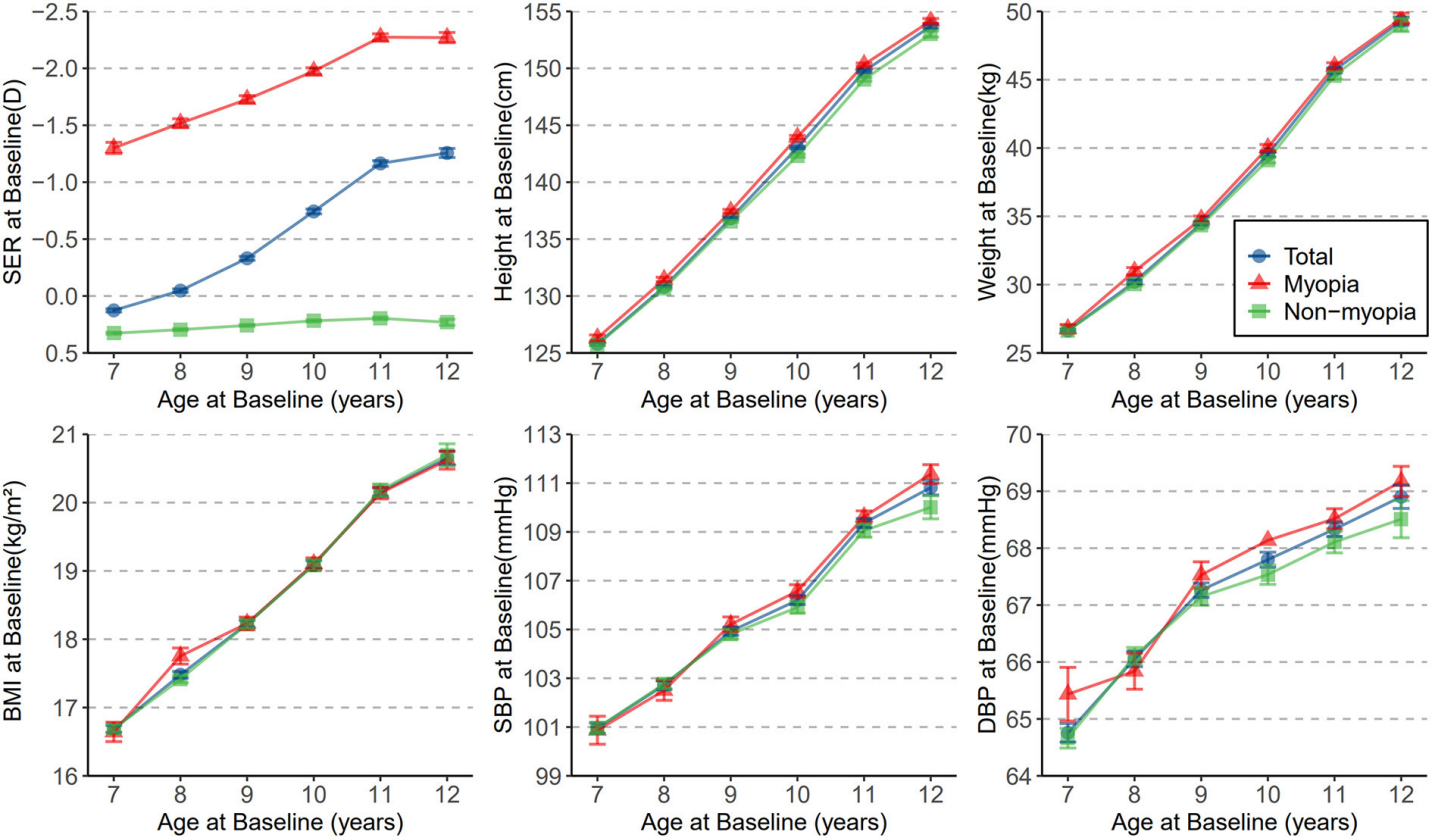
The distribution of mean SER, height, weight, BMI, SBP, and DBP with age at baseline. Dots represents means and whiskers indicate SEM. SER, spherical equivalent refraction; BMI, body mass index; SBP, systolic blood pressure; DBP, diastolic blood pressure.

**Figure 2:**
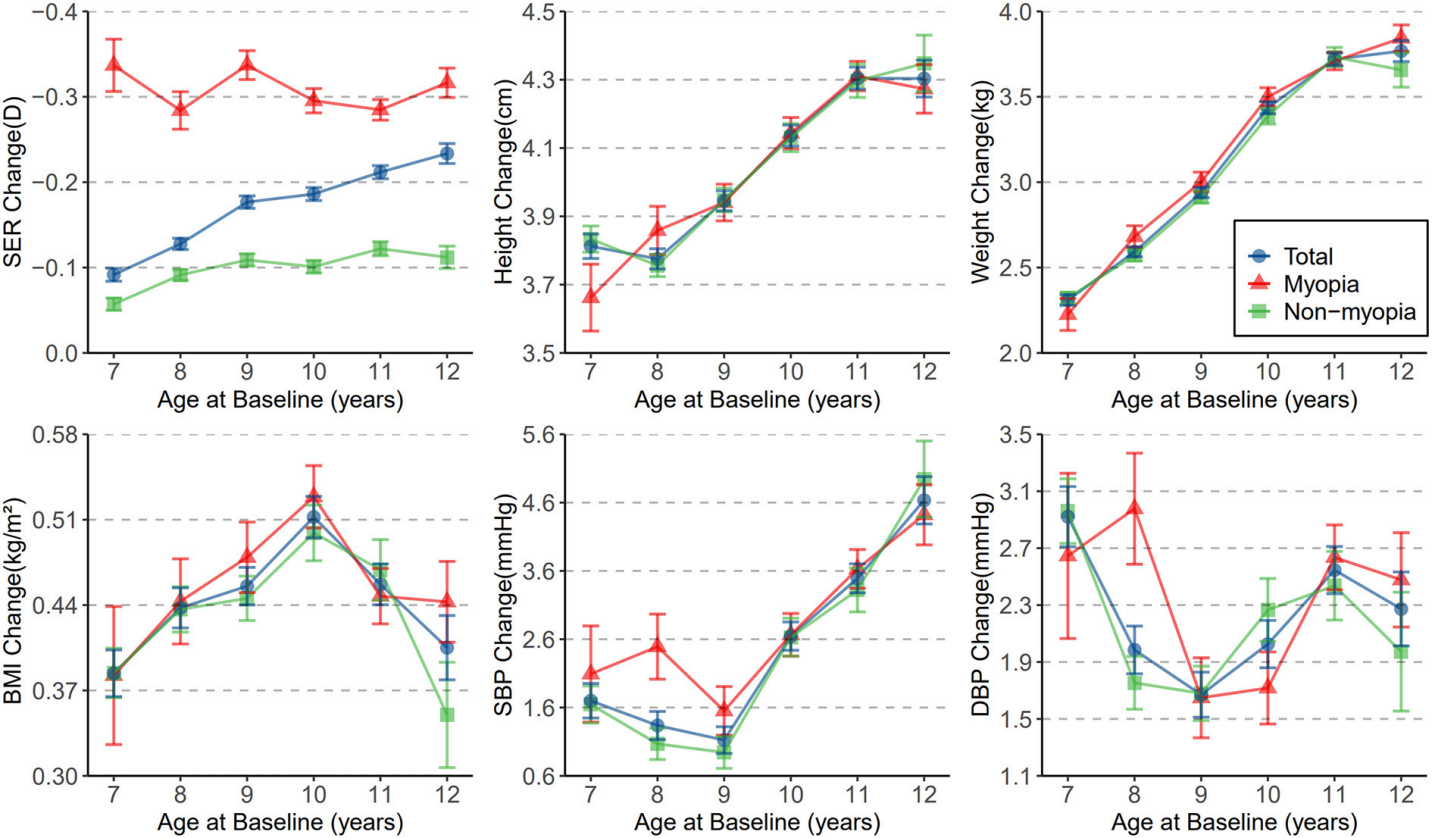
The distribution of mean change in SER, height, weight, BMI, SBP, and DBP with age at baseline. Dots represents means and whiskers indicate SEM.

Figure 1 shows the distribution of mean SERs and mean anthropometric indicators with age at baseline. Some similar trends in participants' negative refraction with each anthropometric indicator. Overall, negative SER and all anthropometric indicators increased with age. Moreover, myopic children showed a significant trend in SER with age than non-myopic children. While myopic children had higher height than non-myopic children at all ages, other anthropometric measures differed significantly at only a few ages (Supplementary Table 1). Figure 2 describes the 6-month mean changes of SER and anthropometric indicators with age. Regarding general trends, the trends in subjects' height and weight and in negative SER were somewhat similar, all increasing with age. Among myopic and non-myopic children, the only significant difference in anthropometric indicators changes was in SBP and DBP at age 8 (Supplementary Table 2).

Bivariate correlations of SER with height, weight, BMI, SBP, and DBP at baseline are shown in Table 2, which was of low to moderate strength. Height, weight, BMI, SBP, and DBP were negatively correlated with SER (*r* = −0.324, *r* = −0.234, *r* = −0.121, *r* = −0.112, *r* = −0.066, both *p* < 0.001). The mean SER by quartiles of height, weight, BMI, SBP, and DBP are shown in Table 3. The taller, heavier, fatter (students with higher BMIs), and higher blood pressure (students with higher SBPs or DBPs) individuals were found to have lower SER and their refractive status tended to be more myopic (*p* < 0.001 for each linear trend test). These results are consistent with the findings for the bivariate correlation of anthropometric indicators with SER. Stratifying the population by refractive status at baseline revealed refractive status based differences between refraction and anthropometric indicators (Table 4). In multiple linear regression models, height, weight, BMI, SBP, and DBP were all correlated with refraction in myopic children. Nonetheless, refraction in non-myopic children is only correlated with height, not weight, BMI, SBP, and DBP.

**Table 2:** Bivariate correlations of SER with height, weight, BMI, SBP, and DBP.

	**SER (D)**			
	**Baseline**		**Changes**	
	***R***	***P*-value**	***R***	***P*-value**
Height (cm)	−0.324	<0.001	−0.034	<0.001
Weight (kg)	−0.234	<0.001	−0.031	<0.001
BMI (kg/m^2^)	−0.121	<0.001	0.000	0.974
SBP (mmHg)	−0.112	<0.001	−0.004	0.509
DBP (mmHg)	−0.066	<0.001	0.007	0.248

Data are the Pearson correlation coefficients.

**Table 3:** Mean values of SER by quartiles of height, weight, BMI, SBP, and DBP.

	**Range**	***n***	**SER (D)**
Height (cm)			
1st quartile	97~131	7,054	0.01 ± 0.98
2nd quartile	132~138	5,914	−0.31 ± 1.19
3rd quartile	139~147	6,481	−0.68 ± 1.48
4th quartile	148~180	6,195	−1.19 ± 1.75
*P* for trend^*^			<0.001
Weight (kg)			
1st quartile	15~27	6,438	−0.07 ± 1.05
2nd quartile	28~34	6,767	−0.39 ± 1.31
3rd quartile	35~43	6,032	−0.67 ± 1.48
4th quartile	44~129	6,407	−0.98 ± 1.70
*P* for trend			<0.001
BMI (kg/m^2^)			
1st quartile	10.56~15.56	6,411	−0.31 ± 1.27
2nd quartile	15.58~17.51	6,428	−0.43 ± 1.34
3rd quartile	17.53~20.85	6,414	−0.60 ± 1.47
4th quartile	20.89~44.37	6,391	−0.76 ± 1.63
*P* for trend			<0.001
SBP (mmHg)			
1st quartile	53~96	6,549	−0.35 ± 1.30
2nd quartile	97~105	6,661	−0.45 ± 1.37
3rd quartile	106~114	6,247	−0.57 ± 0.02
4th quartile	115~180	6,187	−0.74 ± 0.02
*P* for trend			<0.001
DBP (mmHg)			
1st quartile	20~61	7,238	−0.39 ± 1.35
2nd quartile	62~66	5,652	−0.50 ± 1.42
3rd quartile	67~73	6,787	−0.60 ± 1.46
4th quartile	74~128	5,967	−0.63 ± 1.55
*P* for trend			<0.001

Data are presented as the means with SD.

* Tests for linear trend is performed by entering the median value of each category of the anthropometric indicator as a continuous variable in the models.

**Table 4:** Multiple linear regression models of SER by height, weight, BMI, SBP, and DBP for myopic and non-myopic children separately.

	**SER (D)**							
	**Myopic**				**Non-myopic**			
	**Model 1**		**Model 2**		**Model 1**		**Model 2**	
	**B**	***P*-value**	**B**	***P*-value**	**B**	***P*-value**	**B**	***P*-value**
Height (10 cm)	−0.30	<0.001	−0.20	<0.001	−0.04	<0.001	−0.02	0.003
Weight (10 kg)	−0.19	<0.001	−0.11	<0.001	−0.02	<0.001	0.01	0.217
BMI (10 kg/m^2^)	−0.36	<0.001	−0.20	<0.001	−0.01	0.611		
SBP (10 mmHg)	−0.12	<0.001	−0.07	<0.001	−0.01	0.019	0.00	0.809
DBP (10 mmHg)	−0.09	<0.001	−0.06	0.001	0.00	0.718		

Model 1 constructs based on crude data. In model 2 we adjusted for age and gender.

Bivariate correlation analysis showed that SER changes were negatively correlated with height changes (*r* = −0.034, *p* < 0.001) and weight changes (*r* = −0.031, *p* < 0.001), the two correlation coefficients were low but statistically significant. And there was no linear correlation between SER changes and BMI changes (*r* = 0.000, *p* = 0.974), SBP changes (*r* = −0.004, *p* = 0.509) and DBP changes (*r* = 0.007, *p* = 0.248) (Table 2). Table 5 shows that the trend of changes in SER and height, SER and weight were generally consistent, indicating that there is also a significant longitudinal correlation between SER and both height and weight (*p* < 0.001 for each linear trend test). In model 2 of Table 6, growth in height was associated with changes in SER in both groups of children. For every increase in height of 10 cm, SER decreased by 0.12 D (*p* < 0.001) for myopic children and 0.03 D (*p* = 0.035) for non-myopic children. Weight gain in non-myopic but not myopic children is associated with a decrease in SER. Changes in BMI, SBP, and DBP were not correlated with changes in SER in our multiple linear regression model.

**Table 5:** Mean values of SER changes by quartiles of change in height, weight, BMI, SBP, and DBP.

	**Range**	***n***	**ΔSER (D)**
ΔHeight (cm)			
1st quartile	−4~3	10,415	−0.156 ± 0.49
2nd quartile	4	4,893	−0.157 ± 0.55
3rd quartile	5	4,326	−0.171 ± 0.45
4th quartile	6~12	6,010	−0.198 ± 0.55
*P* for trend^*^			*P* < 0.001
ΔWeight (kg)			
1st quartile	−20~2	1,0957	−0.149 ± 0.47
2nd quartile	3	5,112	−0.155 ± 0.50
3rd quartile	4	3,746	−0.188 ± 0.53
4th quartile	5~20	5,829	−0.205 ± 0.57
*P* for trend			*P* < 0.001
ΔBMI (kg/m^2^)			
1st quartile	−12.88~−0.17	6,427	−0.171 ± 0.50
2nd quartile	−0.16~0.43	6,481	−0.167 ± 0.51
3rd quartile	0.44~1.07	6,353	−0.163 ± 0.48
4th quartile	1.08~9.84	6,383	−0.174 ± 0.55
*P* for trend			*P* = 0.825
ΔSBP (mmHg)			
1st quartile	−70~−7	6,469	−0.170 ± 0.49
2nd quartile	−6~2	6,531	−0.181 ± 0.50
3rd quartile	3~11	6,479	−0.149 ± 0.54
4th quartile	12~110	6,165	−0.175 ± 0.52
*P* for trend			*P* = 0.574
ΔDBP (mmHg)			
1st quartile	−62~−5	6,419	−0.180 ± 0.52
2nd quartile	−4~2	6,849	−0.164 ± 0.52
3rd quartile	3~9	6,457	−0.171 ± 0.50
4th quartile	10~106	5,919	−0.160 ± 0.51
*P* for trend			*P* = 0.082

Data are presented as the means with SD.

* Tests for linear trend is performed by entering the median value of each category of the anthropometric indicator as a continuous variable in the models.

**Table 6:** Multiple linear regression models of SER changes by change in height, weight, BMI, SBP, and DBP for myopic and non-myopic children separately.

	Δ**SER (D)**							
	**Myopic**				**Non-myopic**			
	**Model 1**		**Model 2**		**Model 1**		**Model 2**	
	**B**	***P*-value**	**B**	***P*-value**	**B**	***P*-value**	**B**	***P*-value**
ΔHeight (10 cm)	−0.11	<0.001	−0.12	<0.001	−0.04	0.010	−0.03	0.035
ΔWeight (10 kg)	0.01	0.809			−0.06	<0.001	−0.04	0.002
ΔBMI (10 kg/m^2^)	0.10	0.087			−0.04	0.136		
ΔSBP (10 mmHg)	0.00	0.347			0.00	0.625		
ΔDBP (10 mmHg)	0.01	0.091			0.00	0.954		

Model 1 constructs based on crude data. In model 2 we adjusted for age and gender.

## Discussion

Although the peak period of the epidemic has passed, in the post-epidemic era, repeated small-intensity epidemics and the continuous mutation of the virus are still a realistic situation that the public cannot escape (16). Children need to be ready to learn at home for the next wave of the epidemic. We conducted this work because there are few studies on the effects of anthropometric indicators on refraction in children in the post-COVID-19 era. This study found that SER was negatively correlated with height and weight in both cross-sectional and longitudinal analyses. SER was negatively correlated with BMI and blood pressure cross-sectionally but not longitudinally. In addition, refractive status differences were found between the various anthropometric indicators with refraction. The anthropometric and refractive cross-sectional correlations are more robust in myopic children than in non-myopic children, and myopia progresses more rapidly in myopic children than in non-myopic children when they grow to the same height.

AL is an essential indicator of eye growth and is highly correlated with changes in refraction. Many studies have confirmed a significant correlation between height and AL (17, 18). Given that AL is a critical factor in myopia, then there should also be a more significant correlation between refraction and height, but differently, studies on the correlation between height and refraction have not achieved consistent conclusions. A study conducted in Britain on the relationship between height growth trajectory and myopia development found that for each standard deviation increase in height among children aged 2.5–10 years, their SER decreased by 0.075 D and 0.081 D by age 11 and 15 years (19). Another study in Tianjin, China, yielded similar results to us, with the higher the height of the child, the more the refraction tended to be myopic (20). However, Ojami et al. indicated that height is strongly associated with the AL but not with refraction (21).

The AL plays a vital role in refraction, but a long AL does not necessarily mean more severe myopia. Emmetropia is a balance between AL, corneal power and lens power (22). In patients with emmetropes or low myopia, the function of the cornea seems to compensate for the possible myopic effects of slight increases in AL. When increases in AL are excessive, this effect on the cornea tends to disappear (23). In addition, previous studies have proved that the crystalline lens thins during the period of coordinated ocular growth in children, which may also compensate for some of myopia associated with AL growth (24). For these reasons, the relationship between height and refraction may become blurred. Although there is no consensus on the relationship between height and refraction, our study will provide clues for further exploration of the complicated association between the two. In our findings, height and refraction had significant cross-sectional and longitudinal associations under the influence of COVID-19 prevention policies. Therefore, we presume that there may be some common biological regulatory pathways for height and refraction. Several hormones that regulate longitudinal bone growth during childhood have been experimentally demonstrated to play a role in experimental myopia, such as thymic hormones, IGFs, and thyroid hormones (25–27). Additionally, a signaling molecule associated with bone growth, Hedgehog homologs, has also been found to be associated with eye development (28, 29). There is also the view that height and refraction might not be directly related and that they are both independent consequences of increasing socioeconomic status (30, 31). Yet our current study does not include this method of adjusting socioeconomic status.

In a 4-year study, Kearney et al. found that the relationship between height and axial elongation varied by refractive state (5). But the refraction was not included in their analysis. For our analysis, the negative correlation between height and SER was found to be more pronounced in the myopic group. For every 10 cm increase in height, SER shifts 0.3 D toward myopia in myopic children and decreases 0.04 D in non-myopic children. Also, the longitudinal correlation between height and SER was more pronounced in myopic children. How the onset of myopia plays a role in the relationship between height and SER in school-aged children and whether this association is related to the current COVID-19 prevention policy requires further research in the future.

While the impact of obesity on physical health and its association with many systemic diseases is well recognized, little is known about the ocular manifestations of obesity, particularly its impact on refractive development. Weight and BMI correlation with refraction has not been as widely studied as height, and there is no consensus on these correlations. A Burmese-based study shows that heavier individuals tended to be slightly hyperopic (32). In contrast, a study conducted in Korea on young adults claimed no association between weight and refraction (33). In this study, heavier children tended to be myopic in their refraction, and the more they gained weight, the more they reduced their SER. Due to the fact that growth in height usually accompanies an increase in weight, height is essentially positively correlated with weight, which may explain why weight is also negatively correlated with refraction. BMI is independent of height and is a considerably better indicator of obesity than weight. Similar to the results of some cross-sectional studies (34, 35), we found a negative cross-sectional correlation between BMI and refraction but no significant longitudinal correlation. The reason for this is speculated that obese children spend more time in front of TV and computer screens, and they spend less time than recommended on outdoor activities (36). With the mediation effect of outdoor activity time, myopia gradually progresses. Few studies have been conducted on the correlation between blood pressure and myopia. In our study, SBP and DBP were negatively correlated with refraction cross-sectionally but with low correlation coefficients and no correlation longitudinally.

There are some limitations to our study. First, this is a non-cycloplegic photoscreening study. Although spot provides reliable measurements in screening (37, 38), it is not currently considered a substitute for cycloplegic refraction. In China, performing cycloplegic refraction in an extensive sample screening program is a great challenge. Second, part of our subjects with undetected ocular diseases may not have been appropriately excluded from this study due to insufficient information. Given the large sample size of this study, the impact of this limitation on the conclusions should be minimal. Third, the follow-up time is so short that the changes in anthropometric indicators and SER are small among some participants. A longer follow-up period would be necessary to validate our conclusions in further research. Fourth, we did not provide ocular biometrics. Given the strong relationship between refraction and ocular biometrics (39), the association between anthropometric indicators and ocular biometrics should also be considered in further research to explore the complex relationship and mechanisms between anthropometric indicators and refraction.

In conclusion, during the growth of school-age children in the post-COVID-19 era, a significant correlation exists not only between height, weight, BMI, SBP, DBP and refraction but also between height and weight gain and refractive changes. These associations vary by refractive status. It indicates that children's height, weight, and growth rate may be used as reference indicators for myopia risk prediction and progression monitoring. In addition, refractive monitoring of school-aged children should focus on those significantly taller and heavier than their peers.

## Data availability statement

The raw data supporting the conclusions of this article will be made available by the authors, without undue reservation.

## Ethics statement

The studies involving human participants were reviewed and approved by the Ethics Board of Tianjin Medical University Eye Hospital. Written informed consent to participate in this study was provided by the participants' legal guardian/next of kin.

## Author contributions

XQian, WD, and GD designed the study. XG, WD, GD, KA, YW, LW, XL, JL, XQi, YN, LS, and YZ collected participants' data. WD and JL performed the data analysis and participated in manuscript preparation. XQian, NH, and NW revised the manuscript. All authors read and approved the final manuscript.
