# Supplementary material for: Associations between anthropometric indicators and refraction in school-age children during the post-COVID-19 era
Source: Front Public Health. 2023 Jan 18;10:1059465. doi: 10.3389/fpubh.2022.1059465 (PMC9891462; doi:10.3389/fpubh.2022.1059465)
Supplement: Supplementary file 1 [file Table_1.DOCX]

Supplementary Material

# Supplementary Tables

**Supplementary Table 1** P values for anthropometric indicators and refractive comparisons of myopic and non-myopic children in each age group.

| Age | 7 | 8 | 9 | 10 | 11 | 12 |
| --- | --- | --- | --- | --- | --- | --- |
| SER | < 0.001^*^ | < 0.001^*^ | < 0.001^*^ | < 0.001^*^ | < 0.001^*^ | < 0.001^*^ |
| Height | 0.041^*^ | < 0.001^*^ | < 0.001^*^ | < 0.001^*^ | < 0.001^*^ | 0.001^*^ |
| Weight | 0.656 | 0.001^*^ | 0.104 | 0.004^*^ | 0.057 | 0.457 |
| BMI | 0.747 | 0.007^*^ | 0.935 | 0.861 | 0.753 | 0.715 |
| SBP | 0.878 | 0.558 | 0.270 | 0.061 | 0.123 | 0.025^*^ |
| DBP | 0.117 | 0.444 | 0.176 | 0.023^*^ | 0.114 | 0.113 |

T-test for each one.

^*^statistically significant.

**Supplementary Table 2** P values for anthropometric indicators changes and refractive changes comparisons of myopic and non-myopic children in each age group.

| Age | 7 | 8 | 9 | 10 | 11 | 12 |
| --- | --- | --- | --- | --- | --- | --- |
| ΔSER | < 0.001^*^ | < 0.001^*^ | < 0.001^*^ | < 0.001^*^ | < 0.001^*^ | < 0.001^*^ |
| ΔHeight | 0.115 | 0.176 | 0.907 | 0.832 | 0.834 | 0.498 |
| ΔWeight | 0.316 | 0.143 | 0.172 | 0.117 | 0.756 | 0.139 |
| ΔBMI | 0.972 | 0.876 | 0.319 | 0.389 | 0.522 | 0.084 |
| ΔSBP | 0.561 | 0.007^*^ | 0.155 | 0.934 | 0.471 | 0.460 |
| ΔDBP | 0.625 | 0.004^*^ | 0.929 | 0.102 | 0.551 | 0.341 |

T-test for each one.

^*^statistically significant.
